# The impact of paid community health worker deployment on child survival: the connect randomized cluster trial in rural Tanzania

**DOI:** 10.1186/s12913-019-4203-1

**Published:** 2019-07-16

**Authors:** Almamy M. Kanté, Amon Exavery, Elizabeth F. Jackson, Tani Kassimu, Colin D. Baynes, Ahmed Hingora, James F. Phillips

**Affiliations:** 1 Department of International Health, Division of Global Disease Epidemiology and Control, Institute for International Programs, Bloomberg School of Public Health, Johns Hopkins University, Baltimore, MD USA; 2 Ifakara Health Institute, PO Box 78373, Mikocheni, Dar-es-Salaam, Tanzania; 3 Heilbrunn Department of Population and Family Health, Mailman School of Public Health, Columbia University, 60 Haven Avenue, New York City, NY 10032 USA

**Keywords:** Community health worker, Randomized cluster trial, Primary health care, Tanzania, Under five mortality rate, Integrated management of childhood illness, Health and Demographic Surveillance System, Childhood survival rate, Implementation research, Experimental trial

## Abstract

**Background:**

This paper reports on a rigorously designed non-masked randomized cluster trial of the childhood survival impact of deploying paid community health workers to provide doorstep preventive, promotional, and curative antenatal, newborn, child, and reproductive health care in three rural Tanzanian districts.

**Methods:**

From August, 2011 to June 2015 ongoing demographic surveillance on 380,000 individuals permitted monitoring of neonatal, infant and under-5 mortality rates for 50 randomly selected intervention and 51 comparison villages. Over the initial 2 years of the project, logistics and supply support systems were managed by the Ifakara Health Institute. In 2013, the experiment transitioned its operational design to logistical support managed by the Ministry of Health and Social Welfare with the goal of enhancing government operational ownership and utilization of results for policy.

**Results:**

The baseline under 5 mortality rate was 81.3 deaths per 1000 live births with a 95% confidence interval (CI) of 77.2–85.6 in the intervention group and 82.7/1000 (95% CI 78.5–87.1) in the comparison group yielding an adjusted hazard ratio (HR) of 0.99 (95% CI 0.88–1.11, *p* = 0.867). After 4 years of implementation, the under 5 mortality rate was 73.2/1000 (95% CI 69.3–77.3) in the intervention group and 77.4/1000 (95% CI 73.8–81.1) in the comparison group (adjusted HR 0.95 [95% CI 0.86–1.07], *p* = 0.443). The intervention had no impact on neonatal mortality in either the first 2 years (HR 1.10 [95% CI 0.89–1.36], *p* = .392) or last 2 years of implementation (HR 0.98 [95% CI 0.74–1.30], *p* = .902). Although community health worker deployment significantly reduced mortality among children aged 1–59 months during the first 2 years of implementation (HR 0.85 [95% CI 0.76–0.96], *p* = 0.008), mortality among post neonates was the same in both groups in years three and four (HR 1.03 [95% CI 0.85–1.24], *p* = 0.772). Results adjusted for stock-out effects show that diminishing impact was associated with logistics system lapses that constrained worker access to essential drugs and increased post-neonatal mortality risk in the final two project years (HR 1.42 [95% CI 1·07–1·88], *p* = 0·015).

**Conclusions:**

Community health worker home-visit deployment had a null effect among neonates, and 2 years of initial impact among children over 1 month of age, but a null effect when tests were based on over 1 month of age data merged for all four project years. The atrophy of under age five effects arose because workers were not continuously equipped with essential medicines in years three and four. Analyses that controlled for stock-out effects suggest that adequately supplied workers had survival effects on children aged 1 to 59 months.

**Trial registration:**

Registration for trial number ISRCTN96819844 was retrospectively completed on June 21, 2012.

## Background

Each year, over seven million children die before they reach 5 years of age, and over a quarter of one million women die in childbirth [1]. Global monitoring shows that risks of childhood death are highest in sub-Saharan Africa, where four million lives could be saved annually if proven interventions for enhancing maternal, newborn and child survival could reach 90% of families [2]. Tanzania has responded to the challenge that such estimates imply with investment in dispensary-based fixed facility care strategies that have achieved impressive gains in child mortality reduction [3]. However, evidence shows that mortality remains high at all ages of childhood [4] and that maternal mortality reduction has stagnated at 390 per 100,000 live births [5]. Mortality risks to children increase directly with distance to the nearest facility, suggesting that inequitable access has yet to be addressed by health policy and action [6, 7]. The challenge of mortality reduction is particularly evident for newborns, where the mortality rate remains at 26 deaths per 1000 live births, accounting for half of infant mortality and one-third of deaths of children under-5 [8].

Evidence from South Asia has emerged that community health workers (CHW) can accelerate improvement in mortality reduction in resource constrained settings [6, 9]. However, in sub-Saharan Africa, where the need for evidence to justify large scale funding for health systems development is especially acute, few randomized trials have tested the hypothesis that CHW deployment increases child survival [10–12]. More typically, policies promoting CHW deployment are based on plausibility trials [13] or non-experimental demonstration [14, 15]. In Tanzania, volunteer CHW programs in place since the 1970s have been fraught with management and implementation problems [16], and have failed to generate evidence that unpaid workers can be effective or sustainable as providers of primary health care [17]. In 2007, the Government of Tanzania promulgated the Primary Health Services Development Plan, known in Tanzania by its Swahili acronym, MMAM for Mpango wa Maendeleo wa Afya ya Msin. The MMAM policy aimed to revitalize primary health care, although the strategic details for establishing a professional national CHW cadre were unclear [18]. Although implementation of elements of the planned program have since commenced, the CHW component had languished at the time of the onset of Connect owing to a lack of operational clarity about how workers should be recruited, trained and deployed, and whether such workers should be paid. There was no systematic evidence that the deployment of such workers would have an incremental impact, given the ubiquitous dispensary-based program that was already functioning. Connect was intended to address this knowledge gap.

In 2011, the Ifakara Health Institute (IHI), the Tanzania Ministry of Health and Social Welfare, the Tanzanian Training Center for International Health and the Mailman School of Public Health at Columbia University launched a cluster-randomized controlled trial known as “Connect” to evaluate the childhood mortality impact of introducing paid CHWs into local health systems [19]. Connect was a four-year field experiment in Kilombero, Ulanga and Rufiji Districts of Tanzania. Interventions included selection, training, deployment and backstopping of professional CHW and technical assistance to Council Health Management Teams for their provision of essential system supports such as remuneration of the CHW, timely supervision and access to essential medicines and supplies. This paper aimed to assess the childhood mortality impact of paid community-based primary health care workers in rural Tanzania where access to convenient fixed facility care at community dispensaries was already established.

### Setting

**Fig. 1:**
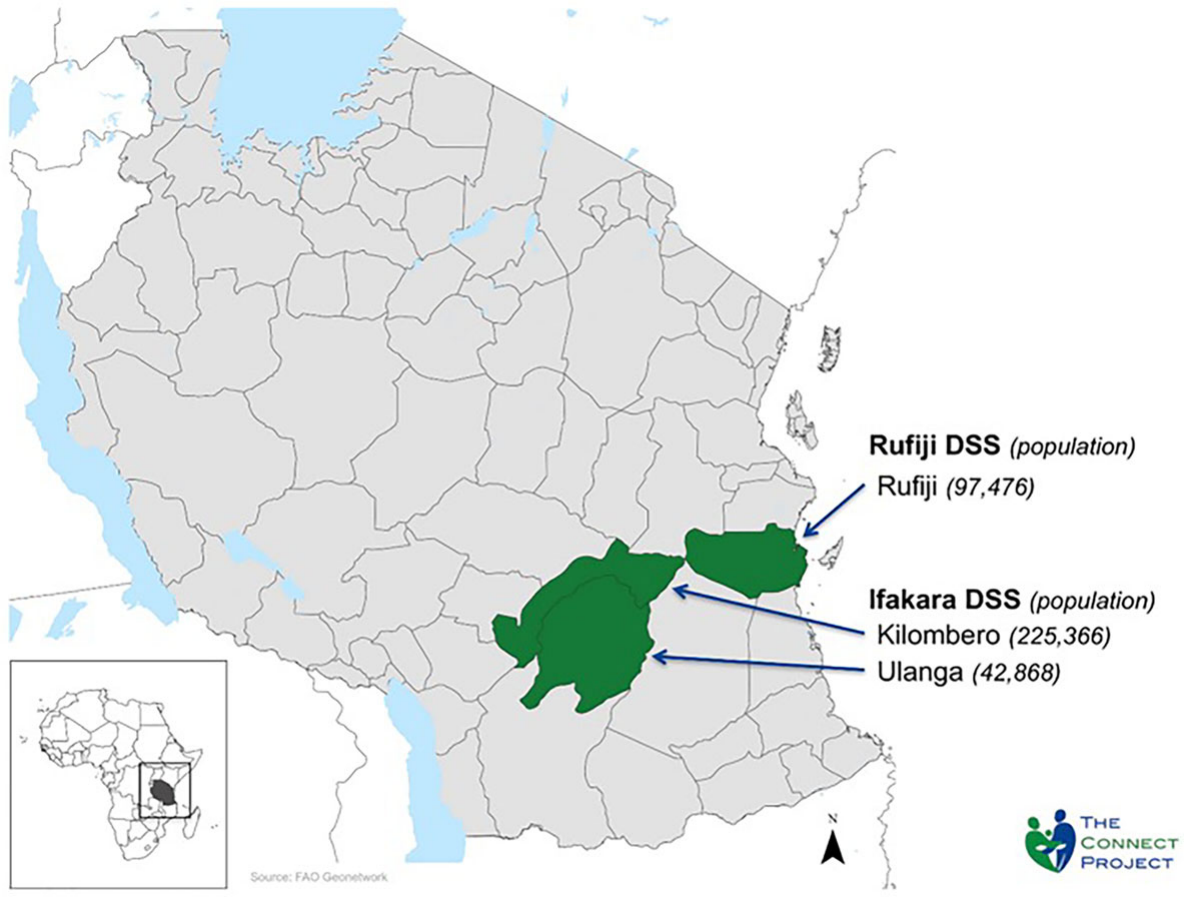
Map of Connect study area, Ifakara and Rufiji Health and Demographic Surveillance Systems (HDSS), population in 2011. Legend: District Baseline HDSS Population; Rufiji 97,496; Kilombero 225,366; Ulanga 42,868

The Connect Project was conducted in study areas of the Ifakara Health Institute that are located in three isolated, rural, and impoverished districts. Research conducted in the Morogoro Region of southwestern Tanzania was located in Ifakara and Ulanga Districts comprising an area that is approximately 500 km by road from Dar-es-Salaam. A study area in the Coast Region, was located in Rufiji District, approximately 150 km south of Dar es Salaam by road. The sites are largely rural with economies that are dominated by subsistence farming, fishing, and petty trading [20, 21]. The population under observation in June 2015 was approximately 380,000 individuals (99,206 in Rufiji and 280,073 in Ifakara). This study population resided in 101 villages (63 in Ifakara and Ulanga Districts and 38 in Rufiji) (Fig. 1). Prior to the Connect Project verbal post mortem investigations determined that causes of childhood mortality in study areas were dominated by malaria (7.8 deaths per 1000 person-years), acute respiratory infections including pneumonia (2.8 deaths per 1000 person-years, and prematurity and low birth weight (1.9 deaths per 1000 person-years), although other preventable causes were prominent, such as diarrheal diseases, birth injuries and asphyxia, anemia, and malnutrition [22].

## Methods

### Study design

Connect was a cluster-randomized trial in geographic clusters covering 101 villages located in Rufiji, Kilombero and Ulanga Districts of Tanzania, where the Ifakara Health Institute operates a Health and Demographic Surveillance System (HDSS) that was adapted from core technology of the international agency known as the International Network of Field Sites with Continuous Demographic Evaluation of Populations and Their Health in Developing Countries (“INDEPTH Network”) [23, 24]. In this approach, a census is taken at the onset of surveillance that defines population composition by age and familial relationships. Subsequent routine interviewer visits update database registers for deaths, births, migration in and out of households, changes in marital status, and establishment of new households [25]. The HDSS has monitored population dynamics in parts of Kilombero and Ulanga Districts since 1996 (Ifakara HDSS) and in half of Rufiji District since 1998 (Rufiji HDSS). At the onset of Ifakara Health Institute HDSS operations, household interviews were conducted every 4 months. Since July 2013 however, household interviews to update database registers have been conducted every 6 months, in a new system that employed automated data editing and calculation of demographic rates.

Stratified randomization techniques were used to allocate 50 villages to the intervention group and 51 to the comparison group. The unit of experimental randomization was the village. In January 2010, a public drawing was organized to randomly assign villages (1:1) to intervention and comparison groups. Villages in the study area differ substantially by population size, location (rural or semi-urban), distance to the nearest health facility and health facility staffing. Stratification was segmented by four categories of village population size to ensure comparability between intervention and comparison areas. Each selected village received between one and four CHWs depending upon population size estimated in 2009. Because of the nature of services provided, it was impossible to mask the participants to their treatment status.

### Objectives

The Connect Project aimed to test the child survival impact of the MMAM policy.

Its operational design aimed to be consistent with national health system structures and requirements, in order to facilitate national scale-up if results warranted such action. Candidate CHWs were required to have completed secondary school grade 10 in order to meet government employment requirements. The recruitment process was managed by Council Health Management Teams with support from the Council Education Officers to review and validate the certificates presented by candidates. Administration of the field operation was a program of the Ministry of Local Government. In this arrangement, the project awarded contracts to Council Health Management Teams, who committed resources to village governments. These local authorities were responsible for hiring, deploying, and compensating CHW. Village governments selected the candidates from a wide pool of potential CHWs who applied. The village assembly selected the finalists. They were required to be residents of the village where they would work. No salary guidelines existed for compensating CHW. However, to maintain equivalence with Government of Tanzania salary guidelines for entry level dispensary workers, all CHW were paid an annual salary in Tanzanian Shillings amounting to US$1348.21 [26].

In compliance with government employment requirements, the duration of CHW training lasted 9 months with the goal of enabling CHW to provide the services that are summarized in Table 1. Training included orientation to human physiology, the treatment and prevention of common ailments, and community organization, as well as a supervised community-based practicum. CHWs were trained in the provision of the WHO recommended integrated management of childhood illness (IMCI) [27, 28] Throughout the implementation period, CHWs provided family planning education, re-supply of oral contraceptives and condoms, sexually transmitted infections/HIV prevention education, safe motherhood and essential newborn care counseling, and provision of IMCI. The IMCI component involved following specific protocols to identify and treat sick children in the initial stages of uncomplicated malaria, pneumonia, or diarrhea. Treatment included antimalarial medicine (Artemether-Lumefantrine, ALu), antibiotics (Co-trimoxazole) and oral rehydration salts (ORS) with zinc sulphate, respectively [29]. CHWs were trained to refer young infants (under 2 months) and children with severe diseases to health facilities [30]. CHWs cooperated with Council Health Management Team planners and service providers in the coordination and household mobilization for under-5 health outreach services in communities, including those that focus on necessary immunizations, vitamin A supplementation and onchocersiasis prevention (Ivermectin). Each month, the CHWs developed a schedule of home visits and community mobilization in accordance with guidance from their supervisors. CHW training also developed core competencies in record keeping, community problem identification, planning, service implementation, monitoring and evaluation, and disease prevention outreach including water, sanitation and hygiene, nutrition, health education, and counseling [19].

**Table 1:** Services provided by CHW for treatment community residents, by age category

Age range in the life cycle:	Services provided by Connect CHW:
Neonates	Essential newborn care: Immediate post partum home visits, Referral for sick children,
	Promotion of postnatal care
	Referral of low birth weight children & follow-up for “kangaroo mothercare.”
Neonates and post-neonates	Immunization of newborns: Tetanus, DPT, BCG,
	Comprehensive expanded program on immunization
Infancy and childhood	Community and doorstep provision of integrated management of childhood illness
	Promotion of safe water, sanitation, and hygiene
Adolescence	Youth friendly reproductive health promotion, including HIV awareness and prevention.
Adulthood	Treatment of common ailments: Malaria, respiratory infections, diarrheal diseases
	Family planning promotion and referral; family planning service provision for condoms and oral contraceptives.
	Health promotion: HIV and sexually transmitted diseases
	Pregnancy and intrapartum care: Pregnancy monitoring and doorstep promotion of antenatal care, skilled delivery, and birth spacing. Emergency referral for acute care needs.
	Promotion of health insurance enrollment, emergency care awareness, and service fee policies.

After the training, CHWs were deployed to their villages as Council Authority employees, remunerated accordingly, and equipped with a mobile phone, bicycle, basic medical supplies and medicines, and service delivery registers. Each CHW was supervised by a Council Health Management Team consisting of a Connect field coordinator, village authorities, and health workers posted in a nearby health facility. Each Council Health Management Team received training in supportive supervision.

Because of training facility availability constraints, CHWs were trained in two batches and deployed in 25 villages in Ifakara and Rufiji HDSS areas in August of 2011 and in 25 remaining villages in August of 2012. Therefore, the start dates of exposure to the program varied by cluster. All clusters that received CHW in 2012 were treated as comparison villages for the period from August 2011 through July 2012. A total of 142 paid CHWs were deployed in the 50 intervention villages. Connect was implemented from August of 2011 through June of 2013 by a project team of the Ifakara Health Institute During this period, CHW services were comprised of household visits and their mobilization of men’s and women’s groups for discussion of specific health topics, with logistics support for essential drugs provided by staff of the Ifakara Health Institute. In 2013, the governing steering committee of the project integrated all logistics support for the project CHW operations into routine mechanisms of the Ministry of Health and Social Welfare. Although this action did not change the experimental design, the operational changes imposed were tantamount to transitioning the project from a randomized cluster trial to an embedded research design [31].

Donor support fully financed project implementation for the first 2 years. During this initial phase, Connect functioned as a primary research trial of the efficacy of CHW deployment. Starting in year 3 of the intervention (July 2013), the implementation of health system support functions, including supervision, logistics, and pharmaceutical supply, was managed by the local government authorities, with technical assistance and financial support for all intervention components, except CHW salaries which were vested in administrative mechanisms of district authorities.

This approach to project management in years 3 and 4 of the project, shifted the trial from an assessment of CHW efficacy to an assessment of the overall system of community-based care effectiveness, in the manner of assuming that the general administration of the scheme was a component of the trial. This effectiveness assessment phase, termed “embedded science,” was intended to maximize integration of project activities into public sector management mechanisms that would ultimately assume responsibility for the large scale utilization of results [31, 32]. Connect staff continuously monitored implementation through field visits and communication and coordination with the Council Health Management Teams responsible for sustaining the flow of resources to communities, and local government officials responsible supervising each CHW.

To ensure health service quality, a dual supervisory system was specified whereby dispensary paramedics maintained oversight of service operations. Local Ministry of Health and Social Welfare dispensary staff also managed the distribution of medicines, supplies and salaries, and continuously recorded implementation problems such as stock-outs of essential medicines and supplies and periods during which salaries were not paid. Meticulous record keeping throughout implementation identifies for each village the months in which the program was or was not implemented according to plan, permitted the imposition of time conditional parameters for the degree of implementation in the statistical analysis of impact.

No paid CHW intervention was planned for the comparison villages. In some comparison villages, there were volunteer CHW and traditional birth attendants who had previously been trained and deployed many years before the trial. Although they did not receive any support from the project during the trial, they may have benefitted from annual vaccination campaigns and may have engaged in some community-based health promotion services. To assess contamination bias, we analyzed the services delivered at the facility level in comparison villages and survey responses. The intervention did not improve the quality and content of care at standard health facilities, as these services were shared by people in both intervention and comparison villages. Health facilities in the study areas were mainly dispensaries with two or three staff members, a consultation room and a basic delivery room. Only one regional hospital was available within the study area in Kilombero. Kilombero also had three health centers and Ulanga had one health center. Rufiji had one private hospital and two health centers. No evidence of contamination bias emerged from analysis of data collected in 2011 and 2013.

### Secondary objectives

Although several measures of morbidity, mortality, health seeking behavior, health service utilization, and program implementation were assessed as specified outcomes, this paper assesses the primary outcome that the Connect CHW program was designed to evaluate: Whether or not the CHW program reduced the under-5 mortality rate, under 5 mortality rate (_5_q_0_), defined as the probability of dying between birth and age-5. Testing the effect of Connect on the neonatal mortality rate and the infant mortality rate (_1_q_0_) are also evaluated.

Data used to derive primary and secondary measures were obtained longitudinally through analysis of HDSS data. Other coverage and maternal and child behavioral outcomes were measured continuously using the HDSS and augmented with additional data from household surveys at baseline and endline. This monitoring included the assessment of a range of health outcomes which are not presented in this paper, including the maternal mortality ratio and adult mortality rates, childhood morbidity, cause of death distribution for children under-five, life years gained, coverage of health services (e.g., rates of antenatal care, skilled attendance at birth, facility delivery, post-natal care, immunization, treatment with oral rehydration solution (ORS), antimalarial medicines, and antibiotics and contraceptive prevalence) the total fertility rate, parental health seeking behaviors during child illness, and other parental health behaviors such as prevalence of immediate and exclusive breastfeeding.

### Data analysis

#### Power calculation

The long legacy of HDSS data provides robust power to assess the effect of the program on childhood mortality. Hayes and Bennett’s formula [33] was utilized to determine the sample size required to detect effects of the intervention on the main health outcome, adjusting for clustering. In response to variable population size across villages, we employed the approach of Van Breukelen [34], and postulated a relative efficiency of their distribution of cluster sizes of 0.85 (relative to equal cluster sizes). HDSS data compiled over the four-year period prior to the intervention were employed to calculate a baseline (2007–2010) under 5 mortality rate of 83.4 deaths per 1000 live births, a coefficient of variation between villages (k) of 0.25, and a mean of 306.8 children-years per cluster. The duration of the experiment was assumed to be an average of 3.1 years of exposure owing to the time that elapsed during CHW deployment phases. The total number of person-years of observation per cluster was 950.9, implying that the study had 80% power to detect a 16% reduction in under 5 mortality rate using a two-tailed test (α = 0.05).

#### Regression analyses

The analysis was a survival-time model with time defined as child’s age in years. Since not all children were followed-up at birth and not all children reached 5 years of age during the experiment, survival times were both left and right censored. Kaplan-Meier analysis was employed to estimate cumulative mortality at 28 days, 1 year and 5 years of age of child. Cox proportional hazard ratios (HR) were estimated to assess the effect of exposure to the intervention on under 5 mortality rate, with robust standard errors to account for clustering. The proportional hazards assumption was tested using Schoenfeld residuals [35]. In circumstances when proportional hazards assumptions were not met (*p* < 0.05), the intervention results were adjusted for age-covariate conditionality [36].

Changes in the risk of dying before age five within the four-year baseline period (pre-intervention: August 2007–July 2011) and the four-year post-intervention period (August 2011–June 2015) were compared between intervention and comparison villages with the use of an adjusted HR (with 95% CI). Adjusted models of pre-intervention mortality outcomes included child age in months to improve precision and calendar year to account for secular mortality trends while controlling for cluster correlation. The adjusted model for post-intervention analysis also took implementation issues into account by including variables measuring the monthly availability of essential medicines for childhood illnesses. Therefore, the intervention group was divided into two subgroups (villages with essentials medicines and villages without).

Analyses were unadjusted for other factors related to the child (gender or birth order), the mother (education attainment, marital status or age group) and village of residence (distance to nearest health facility or socio-economic status) because these variables were excluded from the randomization process for power calculations. Analyses were conducted using the software package Stata version 13.1.

## Results

### Procedures

**Fig. 2:**
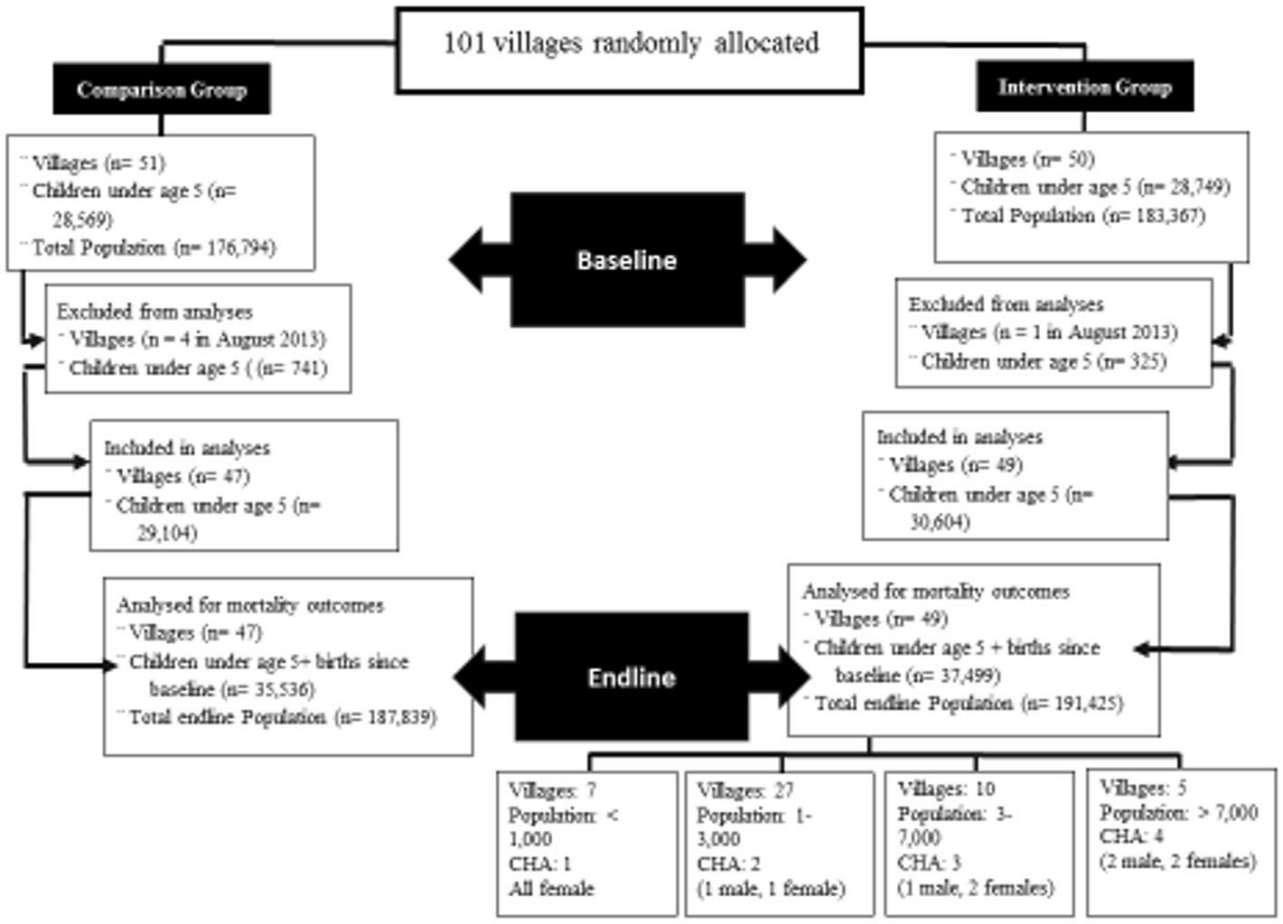
Connect Trial Profile. U5: Population Under age 5, WRA: Population of women of reproductive age, 14–49, CHW: Community Health Worker

A total of the 101 villages were located in HDSS areas, of which 50 were randomly allocated to intervention and 51 to comparison clusters. Owing to financial decisions, surveillance was terminated in five clusters during the study, of which four were comparison clusters and one was a treatment cluster (Fig. 2).

The population of children under-five years old living in the study areas on July 31, 2011 was 30,524 in the intervention group (51.7%) and 28,569 in the comparison group (48.3%). Key demographic characteristics of children, their mothers and households were balanced between intervention and comparison groups (Table 2). The age group and gender of children and the age group and educational attainment of mothers did not differ. The distribution of children by household socioeconomic status was also the same between the two groups.

**Table 2:** Baseline characteristics of the Connect study population

Children observed		*Total*		*Intervention*		*Comparison*	
		number observed	Percent	number observed	Percent	number observed	Percent
Children (0-59monts) characteristics:							
Overall		59,093	100·0	30,524	51·7	28,569	48·3
HDSS	Ifakara	42,929	72·6	21,534	70·5	21,395	74·9
	Rufiji	16,254	27·5	8990	29·5	7264	25·4
District	Kilombero	38,081	64·4	19,252	63·1	18,829	65·9
	Ulanga	4848	8·2	2282	7·5	2566	9·0
	Rufiji	16,254	27·5	8990	29·5	7264	25·4
Sex of child	Boy	29,366	49·7	15,182	49·7	14,184	49·6
	Girl	29,817	50·5	15,342	50·3	14,475	50·7
Age group of child (month)	Less than 1	4472	6·8	2243	6·6	2229	7·0
	1_11	13,796	20·9	6982	20·6	6814	21·2
	12_59	47,747	72·3	24,722	72·8	23,025	71·8
Maternal and household characteristics:							
Age group of mother (years)	< 20	16,209	27·4	8277	27·1	7932	27·8
	20–29	25,710	43·5	13,251	43·4	12,459	43·6
	30–39	14,565	24·6	7583	24·8	6982	24·4
	≥40	2699	4·6	1413	4·6	1286	4·5
Education attainment	Never been to school	32,364	54·8	17,094	56·0	15,270	53·4
	Primary level	25,215	42·7	12,650	41·4	12,565	44·0
	Secondary and plus	1604	2·7	780	2·6	824	2·9
Relative Socio-economic status of household (Wealth Quintiles)	1st Quintile	8469	19·2	4712	20·3	3757	18·0
	2nd Quintile	8700	19·7	4664	20·1	4036	19·3
	3rd Quintile	8936	20·3	5035	21·7	3901	18·7
	4th Quintile	8186	18·6	4254	18·3	3932	18·8
	5th Quintile	7255	16·4	3178	13·7	4077	19·5
	Unknown	2566	5·8	1365	5·9	1201	5·7
Total Population in the study areas:							
Baseline (in July 31, 2011)		360,161	100·0	183,367	50·9	176,794	49·1
Endline (in June 30, 2015)		379,264	100·0	191,425	50·5	187,839	49·5

The baseline under 5 mortality rate was 81.3 deaths per 1000 live births (95% CI 77.2–85.6) in the intervention group and 82.7/1000 (95% CI 78.5–87.1) in the comparison group (adjusted HR 0.99 [95% CI 0.88–1.11], *p* = 0.867). After 4 years of implementation, the under 5 mortality rate was 73.2/1000 (95% CI 69.3–77.3) in the intervention group and 77.4/1000 (95% CI 73.8–81.1) in the comparison group (adjusted HR 0.95 [95% CI 0.86–1.07], *p* = 0.443) (Tables 3 and 4).

**Table 3:** Mortality indicators for intervention and comparison villages, August 2007–June2015, Rufiji, Ulanga and Kilombero districts, Tanzania

Public health impact indicators:	Intervention		Comparison		*p*-value^e^
	N^a^	Est.^b^ (95% CI)	N	Est. (95% CI)	
Baseline August 2007–July2011:^c^					
Neonatal mortality rate	16,609	23·2 (21·0–25·7)	16,195	25·9 (23·5–28·5)	0·129
Post-neonatal mortality rate	23,234	24·9 (23·9–25·8)	22,567	25·8 (24·9–26·8)	0·541
Infant mortality rate	24,220	48·1 (44·9–51·5)	23,569	51·7 (48·4–55·3)	0·134
Post-infant mortality rate	45,752	33·2 (32·3–34·1)	44,636	31·0 (30·1–31·8)	0·245
Under-5 mortality rate (_0_q_5_)	54,086	81·3 (77·2–85·6)	53,058	82·7 (78·5–87·1)	0·673
Endline August 2011–June 2015:^d^					
Neonatal mortality rate	16,230	26·8 (24·3–29·4)	20,266	25·5 (23·4–27·9)	0·632
Post-neonatal mortality rate	22,346	20·9 (20·1–21·7)	29,744	23·0 (22·2–23·8)	0·182
Infant mortality rate	23,237	47·7 (44·4–51·1)	30,984	48·9 (46·0–52·0)	0·575
Post-infant mortality rate	43,506	25·5 (24·9–26·2)	59,173	28·5 (27·8–29·1)	0·084
Under-5 mortality rate (_0_q_5_)	50,879	73·2 (69·3–77·3)	70,098	77·4 (73·8–81·1)	0·134

^a)^N = total population at risk

^b)^Est. = Estimate

^c)^Baseline calculation is based on 4 years before CHW deployment

^d)^The first batch of CHWs was deployed on August 1, 2011 and the duration of exposure to CHW is 4 years·

^e)^Logrank test chi2 of the mortality rate of the classifying variable

**Table 4:** Discrete-time survival models for the effect of Connect experiment intervention versus comparison areas, August 2007–June 2015, Rufiji, Ulanga and Kilombero districts, Tanzania

Mortality Indicator^a^	Baseline versus endline:					
	Baseline (August 2007–July 2011)			Endline (August 2011–June 2015)		
	Hazard Ratio^b^ (95% CI)		*P*-value^c^	Hazard Ratio (95% CI)		*P*-value
Under-5 mortality (0-59 months)	0·99 (0·88–1·11)		0·867	0·95 (0·86–1·07)		0·443
	Exposure period and childhood age specific rates:					
	Early stage (August 2011–July 2013)		Late stage (August 2013–June 2015)		Overall (August 2011–June 2015)	
	Hazard Ratio^b^ (95% CI)	*P*-value^c^	Hazard Ratio (95% CI)	*P*-value	Hazard Ratio (95% CI)	*P*-value
Neonatal mortality (0-28 days)	1·10 (0·89–1·36)	0·392	0·98 (0·74–1·30)	0·902	1·05 (0·85–1·29)	0·659
Post-Neonatal mortality (1-59 months)	0·85 (0·76–0·96)	0·008	1·03 (0·85–1·24)	0·772	0·91 (0·82–1·02)	0·103
Under-5 mortality (0-59 months)	0·92 (0·83–1·03)	0·137	1·01 (0·85–1·20)	0·891	0·95 (0·86–1·07)	0·443

^a)^Source: Rufiji and Ifakara HDSS data, 2016

^b)^Models were adjusted for calendar year

^c)^Standard errors are adjusted for within village cluster effects. Hazard Ratios (HR) for regression models employ dependent variables taking value 1 if the child died during the study period and 0 otherwise

The mortality effect was heterogeneous across children’s age groups and across the years of implementation (Table 4). The intervention had no impact on neonatal mortality in either the first 2 years (HR 1.10 [95% CI 0.89–1.36], *p* = .392) or last 2 years of the study (HR 0.98 [95% CI 0.74–1.30], *p* = .902). Mortality among post neonates was lower during the first 2 years of implementation (August 2011–July 2013) in the intervention group compared to comparison group (HR 0.85 [95% CI 0.76–0.96], *p* = 0.008). In the second half of the study (August 2013–June 2015), mortality among post neonates was the same in both groups (HR 1.03 [95% CI 0.85–1.24], *p* = 0.772).

#### Implementation challenges

**Fig. 3:**
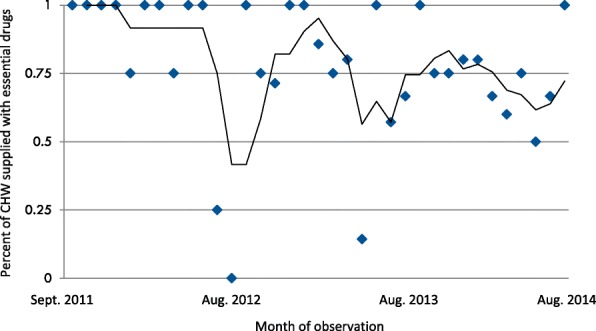
Moving average of the proportion of 50 villages where CHW had access to essential medicines for child illness to treat for uncomplicated pneumonia, malaria and diarrhea by ordinal month following September 2011 (Essential medicines include: Cotrimoxazole or Amoxyciliin, Artemeter Lumefantrine (Alu) and Oral Rehydration Salts and Zinc)

Monthly data on CHW deployment, payment, and stock of essential medicines for childhood illnesses were recorded from September of 2011 through July of 2014. Data comprise 1525 village months of observation because only half of intervention villages (25 out of 50) received CHW during the first year of intervention and because August 2011 data was not collected. During the first year after CHW deployment (September 2011–July 2012), the program was implemented according to protocol and 97.8% (269/275) of intervention villages were supplied with essential medicines. During the second year (August 2012–July 2013), 78.5% (471/600) of the intervention villages had a CHW with supplies, while it was only 75.5% (453/600) in year 3 (August 2013–July 2014). In the final year (August 2014–June 2015), only 1 month (August 2014) had been collected and 62.0% (31/50) of the intervention village had a CHW with supplies (Fig. 3).

#### Differences in impact by period and CHW supply of essential medicines

When the phase 1 HR is assessed taking the presence of essential medicines into account in those villages and months where medicines were present, the adjusted HR for post-neonates was 0·85 (95% CI 0·75–0·96, *p* = 0·012) comparing mortality in those villages whose CHW had essential medicines in particular months with comparison area villages, and the adjusted HR was 0·85 (95% CI 0·68–1·04, *p* = 0·115) comparing mortality in those villages whose CHW did not have essential medicines in particular months with the comparison area. The adjusted HR for neonates was 1·11 (95% CI 0·88–1·41, *p* = 0·385) comparing mortality in those villages whose CHW had essential medicines in particular months and it was 1·03 (95% CI 0·72–1·48, *p* = 0·860) comparing mortality in those villages whose CHW did not have essential medicines in particular months with the comparison area.

During phase two, the adjusted HR for post-neonates was 0·95 (95% CI 0·79–1·14, *p* = 0·589) in the months when a CHW had essential medicines and it was 1·42 (95% CI 1·07–1·88, *p* = 0·015) in the months when a CHW did not have essential medicines. The adjusted HR for neonates was 1·01 (95% CI 0·75–1·34, *p* = 0·957) in the months when a CHW had essential medicines and it was 0·89 (95% CI 0·59–1·33, *p* = 0·562) in the months when a CHW did not have essential medicines.

## Discussion

The Connect trial aimed to test the effectiveness of child survival and reproductive health impact of a package of community-based preventive, promotional, and curative services provided by professional CHW. Results show that the package of capabilities, worker deployment, and technologies comprising interventions did not significantly reduce under-5 mortality in rural Tanzania as an average treatment effect compiled over the entire four-year period of observation. Mortality among children aged 1–59 months declined in the intervention group, but relative to levels observed in the control communities, the effect size was not significant. This null result is comparable to findings reported from trials in Burkina Faso, Ethiopia, Sierra Leone, Uganda (Central) and Uganda (Western) [37].

Efficacy of CHW deployment was nonetheless evident during the early period of the intervention (years 1 and 2) when CHW were supplied with essential medicines by the logistics systems of the Ifakara Health Institute. Such findings are comparable to a Gambia study that showed a significant reduction in child mortality during the initial period (1989–1994) when CHW programs were strongly supported by the government, donor agencies, and villagers who were served by the program. As project implementation progressed over the 1994 to 1996 period, there was diminishing direct support for the project from the research team, and concomitant expansion of political and financial support for the program by routine logistics operations of the government. This shift was associated with an atrophy of impact [38].

Despite the null result of CHW exposure as a Connect project treatment systems effect, initial randomized cluster trial cell comparisons lend support to a growing consensus that CHW provision of primary health care has significant value [9, 39], if workers are adequately supported. In the high mortality context of rural Tanzania, improving access to basic primary health care reduces childhood mortality. Results show, however, that the magnitude of these effects increases as childhood age advances, owing to the efficacy of community-based care for treatment of infectious disease morbidity. Moreover, as the observation time of the project progressed, the impact of CHW exposure declined, because the integrity of logistics and supply implementation atrophied over time, offsetting the potential impact of CHW on the treatment of childhood illness.

Although birth practices and delivery care are fundamentally important to the survival of neonates, infectious disease morbidity is relatively less pronounced among neonates than among older children. CHW had no apparent effect on neonatal survival, a result that is similar to a study in Ghana [40], but contrasting with results of South Asian studies reporting that CHW can improve newborn survival [11]. Although the project promoted the referral of pregnant women and their sick children in both treatment and comparison communities – by repairing seven ambulances and buying one ambulance and two boat-ambulances for health centers and hospitals – the rugged and isolated terrain of the study area, along with the poor quality of clinical services, may have constrained the efficacy of these actions in improving newborn survival. CHW were trained to refer low-birth-weight and ill newborns to health facilities where the project lacked interventions for improving Table 5 the quality of facility-based care.

**Table 5:** The conditional effect of CHW access to essential medicines on neonatal, early childhood and under-5 mortality by stage in the Connect Project, August 2011–June 2015, Rufiji, Ulanga and Kilombero districts, Tanzania

Mortality Indicator^a^	Early stage (August 2011–July 2013)			
	CHW without Medicines		CHW with Medicines	
	HR^b^ (95% CI)	*P*-value^c^	HR (95% CI)	*P*-value
Neonatal mortality (0-28 days)	1·03 (0·72–1·48)	0·860	1·11 (0·88–1·41)	0·385
Post-Neonatal mortality (1-59 months)	0·85 (0·68–1·04)	0·115	0·85 (0·75–0·96)	0·012
Under-5 mortality (0-59 months)	0·90 (0·73–1·11)	0·329	0·93 (0·83–1·04)	0·181
	Late stage (August 2013–June 2015)			
Neonatal mortality (0-28 days)	0·89 (0·59–1·33)	0·562	1·01 (0·75–1·34)	0·957
Post-Neonatal mortality (1-59 months)	1·42 (1·07–1·88)	0·015	0·95 (0·79–1·14)	0·589
Under-5 mortality (0-59 months)	1·21 (0·95–1·55)	1·125	0·97 (0·82–1·16)	0·763
	Overall (August 2011–June 2015)			
Neonatal mortality (0-28 days)	0·98 (0·73–1·31)	0·898	1·06 (0·85–1·32)	0·592
Post-Neonatal mortality (1-59 months)	1·01 (0·82–1·24)	0·933	0·90 (0·80–1·00)	0·054
Under-5 mortality (0-59 months)	0·99 (0·85–1·18)	0·979	0·95 (0·84–1·07)	0·384

^a)^Source: Rufiji and Ifakara HDSS data, 2016

^b)^Models were adjusted for calendar year

^c)^Standard errors are adjusted for the clustering of villages. Hazard Ratios (HR) for regression models of child mortality employ dependent variables taking value 1 if the child died during the study period and 0 otherwise

^d)^Essentials medicines are Co-trimoxazole or Amoxyciliin, Artemeter Lumefantrine (ALu) and Oral Rehydration Salts and Zinc to treat for uncomplicated Pneumonia, Malaria and Diarrhea

There was no differential effect of the CHW program on mortality by child gender, mother’s education attainment, or quintiles of wealth of the household. This Connect result contrasts with findings from experimental studies from India that showed reduction in health inequalities between sub-groups [41].

Questions concerning the efficacy of CHW as health promoters who are not health care providers were not the subject of the Connect design and had no direct bearing on its randomization. However, the atrophy of impact that arose when workers lacked access to supplies suggests that curative functions for CHW are essential to achieving significant child survival gains from their deployment. In fact, evidence from years three and four of Connect suggests that CHW deployment without concomitant provision of supplies may cause more harm than good. The adjusted late stage HR reported in Table 5 is estimated to be 1.42 (95% CI 1·07–1·88, *p* = 0·015). The cause of this effect merits careful investigation. Studies elsewhere have shown that deployment of health volunteers who are not equipped to provide care can foster delay in parental health seeking with detrimental survival consequences [42]. This possible detrimental outcome attests to the need for implementation research on support systems for CHW operations, possibly in the form of transfer experiments that would test the replicability of phase one strategies in locations geographically removed from IHI research sites. Such phased research in other settings has demonstrated the value of separating implementation research from primary impact research to enhance the operational ownership of implementation and clarify the organizational requirements of scaling up strategies that experimental programs have proven to work [43, 44].

### Study limitations

#### Our study had noteworthy limitations

Program operations may not have controlled for contamination that could arise from non-governmental organizational activities in study areas. It is known, for example, that volunteers were deployed in some communities by local non-governmental agencies. While these workers were not trained, supplied, or equipped to provide preventive or curative care, their health promotion activities may have offset potential program effects. While most such activity occurred in comparison cluster communities, the numbers of volunteers and the precise nature of their activities was not factored into the analysis.

Although households located in the comparison communities were unexposed to project CHW deployment, the possibility remains that comparison community parents could seek care for sick children from Connect CHW because villages in intervention and comparison were interspersed without boundaries or barriers that would prevent parents from seeking care from CHW in neighboring treatment communities. CHW were instructed by project field coordinators not to provide services in the comparison communities, but were allowed to provide care to any parent who traveled to treatment areas to seek their care.

Training and deployment of CHW may have affected clinical worker performance in ways that contributed to the quality of care. However, analysis of monitoring systems data have determined that indicators of the quality of services, such as access to health facilities, personnel, and supplies in intervention and comparison groups was comparable throughout the intervention period.

HDSS data were compiled through home visits in all 101 clusters that were conducted in 4 months intervals until June 2013, and in 6 months thereafter. The Connect monitoring team relied upon HDSS data collectors to interview participants about pregnancy status, pregnancy outcomes, and child deaths. From July 2013 to December 2014, the research team audited the HDSS data and found that the field interviewers did not visit approximately 10% of households according to scheduled activities. However, this missing information was distributed equivalently among the intervention and comparison groups. HDSS leaders and field managers were informed and data were collected retrospectively to fill any such interviewing gaps that arose. However, possibility remains that some missing under-five deaths, in particular early newborn deaths, were not captured.

Owing to budgetary constraints and administrative decisions that were unrelated to Connect, HDSS data collection was terminated in July 2013 in five non-randomized Rufiji District villages of which one was a treatment village and four were control villages. Although CHW posted in treatment villages continued operations throughout the project, and data analysis included information for the excluded villages until the onset of censoring in July of 2013, the possible bias that this truncation may have introduced is unknown.

The Connect Project was designed as a blend of embedded implementation science with a formal RCT. This design attempted to maximize prospects that results would be utilized for policy, without the delays and costs that would arise if a separate phase in the research were required to test replicability of project results. While this focus on embedding research into routine operations was intended to position results for national scale-up, the blending of embedded science with an RCT design had the unintended effect of shifting the focus of observation from assessing the demographic impact of community exposure to functioning CHW care to focusing instead on clarifying operational procedures, methods, and milestones required for the effective post-experimental utilization of the project strategic design. The duration interventions, spanning only 3.1 years, with the embedded science paradigm applying to the final half of program exposure, subjected Connect to lapses in logistics and supply operations that were caused, in part, by local government and Ministry of Local Government managerial inexperience with community-based primary health care.

A longer observation time would also be required for the appraisal of the full range of benefits that could accrue from modalities that Connect CHW dispensed. Moreover, since non-specific benefits of vaccination and micro-nutrient supplementation, are known to require multi-year exposure time for full effects to arise [45–48], the limited project exposure time may have constrained prospects for CHW to have their full impact.

Despite these limitations, our findings attest to the potential value of CHW in reducing child mortality. These results in Africa thus confirm evidence emerging from trials in South Asia. However, project results also attest to the importance of ensuring integrity of support systems that enable CHW services to work. The contrast of phase two results with phase one findings challenges the concept of transferring research-managed operations to authorities who will be responsible for scaling up Connect. Transfer experiments require designs that focus on implementation endpoints and organizational processes [49].

## Conclusion

Tanzania is currently engaged in a process of scaling up the CHW model that Connect has tested, but in pursuing this policy, there is a critical need to guide the scaling-up process with implementation research on the operational requirements of sustaining essential support systems. Moreover, systems perspectives on community-based primary health care are essential so that trials include a focus on the quality of fixed facility care and referral capabilities. If the deployment of CHW alone is the focus of intervention, without adequate attention to logistics support requirements, the high burden of newborn morbidity and mortality will persist. Ensuring the effective functioning of referral systems, and improved quality of facility-based care and skilled delivery services are essential elements of an effective context for primary health care that includes the addition of CHW. Connect has demonstrated that CHW can induce childhood mortality decline if CHW deployment is pursued in conjunction with concomitant attention to the effective implementation of essential logistics support systems.

## Data Availability

Africa Health Initiative coordination mechanisms sponsored by the Doris Duke Charitable Foundation supported a Data Cooperative for organizing international access to Africa Health Initiative project data sets. Data of the Connect Project are described at https://www.jhsph.edu/research/centers-and-institutes/institute-for-international-programs/_documents/phit_concept.pdf. Core indicators for this study are displayed at: https://dataverse.harvard.edu/file.xhtml?persistentId=doi:10.7910/DVN/26539/PN40RD. For access to individual level statistical commands, readers are advised to communicate with the corresponding author or with the Ifakara Health Institute Evaluation Thematic Group led by Dr. Eveline Geubbels, at [egeubbels@ihi.or.tz]. Unrestricted access to demographic surveillance data is available through the data repository located at www.indepth-ishare.org and the IHI data portal [http://data.ihi.or.tz/index.php/catalog/central].

## Abbreviations

CHW: Community Health Worker
CI: Confidence interval
HDSS: Health and Demographic Surveillance System
HIV: Human immunodeficiency virus
HR: Hazard Ratio
IHI: Ifakara Health Institute
IMCI: Integrated management of childhood illness
INDEPTH: International Network of Field Sites with Continuous Demographic Evaluation of Populations and Their Health in Developing Countries
MMAM: Swahili acronym Mpango wa Maendeleo wa Afya ya Msingi, English translation: Primary Health Services Development Plan
ORS: Oral rehydration solution
